# Functional expression, purification, and biochemical properties of subtilase SprP from *Pseudomonas aeruginosa*

**DOI:** 10.1002/mbo3.275

**Published:** 2015-07-14

**Authors:** Alexander Pelzer, Christian Schwarz, Andreas Knapp, Astrid Wirtz, Susanne Wilhelm, Sander Smits, Lutz Schmitt, Horst Funken, Karl-Erich Jaeger

**Affiliations:** 1 Institute of Molecular Enzyme Technology, Heinrich-Heine-University Duesseldorf, Forschungszentrum JuelichD-52426, Juelich, Germany; 2 Institute of Biochemistry, Heinrich-Heine-University DuesseldorfUniversitaetsstr. 1, D-40225, Duesseldorf, Germany; 3 Institute of Bio- and Geosciences IBG-1: Biotechnology, Forschungszentrum Juelich GmbHD-52426, Juelich, Germany; 4 Present address: BRAIN AGD-64673, Zwingenberg, Germany; 5 Present address: Bayer Pharma AGD-51373, Leverkusen, Germany

**Keywords:** Autocatalytic processing, heat induction, *Pseudomonas aeruginosa*, subtilase SprP.

## Abstract

The *Pseudomonas aeruginosa* genome encodes a variety of different proteolytic enzymes several of which play an important role as virulence factors. Interestingly, only two of these proteases are predicted to belong to the subtilase family and we have recently studied the physiological role of the subtilase SprP. Here, we describe the functional overexpression of SprP in *Escherichia coli* using a novel expression and secretion system. We show that SprP is autocatalytically activated by proteolysis and exhibits optimal activity at 50°C in a pH range of 7–8. We also demonstrate a significant increase in *sprP* promoter activity upon growth of *P. aeruginosa* at 43°C indicating a role for SprP in heat shock response.

## Introduction

Proteases represent an important class of enzymes found in all kingdoms of life and they are often involved in controlling regulatory cascades by processing and degradation of proteins (Gottesman 2003; Gur et al. 2011). Furthermore, proteases can be part of regulatory processes like the protein quality control (Hengge and Bukau 2003). In *Pseudomonas aeruginosa*, proteases with regulatory function are involved in motility, biofilm formation, antibiotic resistance, and virulence (Brazas et al. 2007; Breidenstein et al. 2012; Fernandez et al. 2012). Several extracellular proteases serve as potent virulence factors (Haas 2003; Hoge et al. 2010; Pearson et al. 2011; Kida et al. 2013; Tang et al. 2013) and have been characterized extensively, including alkaline protease (Kharazmi 1991), protease IV (O'Callaghan et al. 1996; Engel et al. 1998), small protease (Marquart et al. 2005; Tang et al. 2013) and elastase (Suter 1994; Preston et al. 1997).

The serine protease family S8 harbors the endopeptidase subtilisin and related enzymes with a characteristic Asp/His/Ser catalytic triad (Siezen and Leunissen 1997; Rawlings et al. 2010). The members of this family are called “subtilisin-like serine proteases” or “subtilases” and represent the second largest family of serine proteases (Rawlings et al. 2012). These subtilases often consist of a multi-domain structure composed of a signal sequence, a domain that acts as an intramolecular chaperone and the Peptidase S8 domain (Siezen et al. 1991; Li et al. 1995; Siezen and Leunissen 1997).

The genome of *P. aeruginosa* encodes two subtilases (Winsor et al. 2011), the autotransporter EprS presumably involved in virulence (Kida et al. 2013), and the subtilase SprP affecting a variety of cellular processes (Pelzer et al. 2014). SprP contains 590 amino acids forming three distinct domains, namely a signal sequence, a domain of unknown function (DUF), and a Peptidase S8 domain (Pelzer et al. 2014). Here, we describe the heterologous production and biochemical characterization of SprP. Furthermore, we could demonstrate that the promoter activity of *sprP* is strongly induced at 43°C indicating that SprP may play a role in the heat shock response of *P. aeruginosa*.

## Materials and Methods

### Bacterial strains, media, and culture conditions

*Escherichia coli* DH5*α* was used as host for cloning and was grown in lysogeny broth medium (10 g/L tryptone, 5 g/L yeast extract, 10 g/L NaCl) at 37°C. NEB Express Competent *E. coli* (New England Biolabs, Frankfurt on the Main, Germany) were used for expression of *sprP*-*hlyA1*. Cultures were grown in 2× yeast extract and tryptone (YT) medium (16 g/L bacto-tryptone, 10 g/L bacto-yeast extract, 5 g/L NaCl, 5 mmol/L CaCl_2_, pH 7.2) at 37°C.

### Recombinant DNA techniques and gene cloning

Recombinant DNA techniques were performed essentially as described by Sambrook et al. (1989). DNA fragments were amplified by PCR standard methods. DNA modifying enzymes (Thermo Scientific, Darmstadt, Germany) were used according to the manufacturer's instructions. Plasmid DNA was prepared by using the innuPREP Plasmid Mini Kit (Analytik Jena, Germany) or, for genomic DNA from *P. aeruginosa*, the DNeasy Blood & Tissue Kit (Qiagen, Hilden, Germany).

### Construction of SprP expression plasmid

The In-Fusion HD Cloning Kit (Takara Bio Europe/Clontech, Saint-Germain-en-Laye, France) was used in accordance to the user manual to construct pSU-SprPminLS. Briefly, the In-Fusion enzyme fuses DNA fragments for example, PCR fragments and linearized vectors by recognizing a 15 bp overlap at their ends. Here, the plasmid pSU-HlyA1 served as vector and was linearized with primer pSUHlyA1_lin_XaHis_for and Primer pSU-HlyA1_lin_rev (Table1). Template DNA was removed by *Dpn*I digestion. The *sprP* gene was amplified by primers SprPminLS_fw and SprP_XaHis_rev with 15 bp extensions homologous to the ends of the vector ends. The linearized vector and the amplified *sprP* gene were incubated with the In-Fusion enzyme resulting in the insertion of *sprP* in the vector pSU-HlyA1 in front of *hlyA1*.

**Table 1:** Strains, plasmids, and primers

Strain or plasmid	Genotype/phenotype	Reference or source
Strains		
*Pseudomonas aeruginosa*		
PAO1	Wild type	Holloway et al. (1979)
*Escherichia coli*		
DH5*α*	*fhuA2Δ(argF-lacZ)U169 phoA glnV44 Φ80Δ (lacZ)M15 gyrA96 recA1 relA1*endA1 thi- 1 hsdR17	Woodcock et al. (1989)
NEB Express Competent	*fhuA2 [lon] ompT gal sulA11 R(mcr-73::miniTn10–TetS)2 [dcm] R(zgb-210::Tn10–TetS)*endA1 Δ(mcrC-mrr)114::IS10	
Plasmids		
pSU-SprPminLS	sprP gene in pSU-HlyA1	This work
pSU-HlyA1	Apr	Schwarz et al. (2013)
pk184-HlyBD	Kmr	Bakkes et al. (2010)
pTZsprP	Cbr; sprP-lacZ fusion	Pelzer et al. (2014)
Primer for cloning	(5′–3′)	
pSUHlyA1_lin_XaHis_for	ATTGATGGCCGTCACCACCACCACCACCACGGAAATTCTCTTGCAAAAAATGTATTA	
pSU-HlyA1_lin_rev	CATTTAATTACCTCTTAACCAGTTAATG	
SprPminLS_fw	AGAGGTAATTAAATGGCCGAAACACCCCTG	
SprP_XaHis_rev	GTGACGGCCATCAATGCGCACGCGCTC	

### Production and purification of SprP

About 100 mL NEB Express Competent *E. coli* cells harboring plasmid pSU-SprPminLS and pk184-HlyBD (Bakkes et al. 2010) were grown in 2× YT medium at 37°C supplemented with ampicillin (100 *μ*g mL^−1^) and kanamycin (30 *μ*g mL^−1^). At an OD_580_ of 0.6, expression was induced with 1 mmol/L isopropyl-*β*-D-thio-galactopyranoside. After 6 h growth, cultures were centrifuged for 30 min at 5000*g* (4°C) and the resulting supernatant was used for SprP purification. The supernatant was concentrated to 1 mL by using Amicon Ultra-15 Centrifugal Filter Units with a 50 kDa cut off (Merck KGaA, Darmstadt, Germany) and washed three times with 10 mL incubation buffer (10 mmol/L Tris-HCl, 300 mmol/L NaCl, 5 mmol/L CaCl_2_, pH 8, 4°C). Five milliliter of this solution was incubated with 1 mL Ni-NTA agarose (Qiagen, Hilden, Germany) for 3 h at 4°C, afterwards loaded on a chromatography column and washed with washing buffers 1 and 2 (washing buffer 1/2: 10 mmol/L Tris-HCl, 300 mmol/L NaCl, 5 mmol/L CaCl_2_, 20/30 mmol/L imidazole, pH 8, 4°C). The SprP-HlyA1 fusion protein was eluted by 5 mL elution buffer (10 mmol/L Tris-HCl, 300 mmol/L NaCl, 5 mmol/L CaCl_2_, 250 mmol/L imidazole, pH 8, 4°C). The elution fraction was concentrated to 0.5 mL using Amicon Ultra-4 Centrifugal Filter Units with a 50 kDa cut off (Merck KGaA, Darmstadt, Germany) and washed three times with 5 mL storage buffer (200 mmol/L Tris-HCl, 5 mmol/L CaCl_2_, pH 8, 8°C) to obtain a final volume of 1 mL. All purification steps were performed at 4°C. The protein concentration was determined with the Qubit 2.0 Fluorometer (Invitrogen, Darmstadt, Germany).

### Determination of protease activity

SprP protease activity was determined with resorufin-labeled casein (Roche, Mannheim, Germany) as the substrate according to the manufacturer's instructions. Three microgram of SprP was incubated at 40°C for 2 h in the presence of the substrate in 200 mmol/L Tris-HCl buffer pH 8 containing 5 mmol/L CaCl_2_. The absorbance of released resorufin-labeled peptides was measured at 574 nm. SprP activity was determined at different temperatures; pH-dependency of SprP activity was determined in Britton-Robinson buffer at 40°C (Britton and Robinson 1931).

Casein gel zymography was used for the detection of proteolytic activity after electrophoresis. Novex 4–16% Zymogram (Blue Casein) Protein Gels (Invitrogen, Darmstadt, Germany) were used according to the manufacturer's instructions. After electrophoresis, the proteins were renatured in Novex Zymogram Renaturing Buffer (Invitrogen, Darmstadt, Germany) and the gel was incubated for 16 h at 37°C in Novex Zymogram Developing Buffer (Invitrogen, Darmstadt, Germany). Protease activity is visible as clear bands against a dark background.

For determination of protease inhibition, 4-(2-aminoethyl)benzenesulfonyl (AEBSF), *N*-*p*-tosyl-l-phenylalanine chloromethyl ketone (TPCK), ethylenediaminetetraacetic acid (EDTA), pepstatin A, or E-64 were added to the reaction tube as described by the manufacturer (Sigma-Aldrich, Seelze, Germany) and incubated for 1 h at 4°C. Afterward, protease activity was determined with resorufin-labeled casein as described above.

### SDS-PAGE

Proteins were separated by sodium dodecyl sulfate polyacrylamide gel electrophoresis (SDS-PAGE) under denaturing conditions in a discontinuous gel system (Laemmli 1970). Prior to SDS-PAGE, the protein samples were suspended in SDS-PAGE sample buffer, boiled for 10 min at 99°C and loaded onto a 12% polyacrylamide gel. The electrophoresis was run for 15 min at 100 V to concentrate and for 60 min at 150 V to separate the proteins. After electrophoresis, the proteins were stained with Coomassie Brilliant Blue R250 (Neuhoff et al. 1988).

### Protein precipitation

Proteins were concentrated by precipitation using trichloroacetic acid (TCA) (Peterson 1977, modified). The sample was mixed with 1/10 volume of a 1% (w/v) sodium dodecyl sulfate solution and incubated for 10 min at room temperature. Afterward, the sample was mixed with 1/10 volume of a 70% (w/v) TCA solution and incubated on ice for 10 min. The sample was centrifuged for 30 min at 21,000*g* and the sedimented proteins were washed with 500 *μ*L 80% (v/v) acetone cooled to −20°C. The protein pellet was dried and analyzed by SDS-PAGE.

### SprP promoter activity assay

*Pseudomonas aeruginosa* PAO1 was transformed with plasmid pTZ*sprP* and grown at 37°C as well as at 43°C and promoter activity of *sprP* was monitored as previously described (Pelzer et al. 2014).

### Substrate specificity of SprP

A PepSets REPLi (Mimotopes, Notting Hill, Australia) peptide library consisting of 3375 peptides with a variable tripeptide core and a terminal FRET (fluorescence resonance energy transfer) pair was used to screen for substrate specificity. After cleavage of a tripeptide core by SprP, the emission at *λ*_420_ was detected after excitation at *λ*_320_ using an Infinite M1000 PRO photometer (Tecan, Maennedorf, Switzerland). Peptides were dissolved according to manufacturer's instructions in 45 *μ*L of reaction buffer (200 mmol/L Tris-HCl, pH 8, 5 mmol/L CaCl_2_), 2 *μ*g of SprP was added per well (total reaction volume: 100 *μ*L) and the fluorescence was determined after 24 h. Hydrolyzed peptides were tested again by adding 4 *μ*g SprP per well and determination of fluorescence at 2 min intervals for 1 h at 37°C. The activity of SprP was calculated using the linear range of fluorescence increase over time and the sequences of the tripeptides which were hydrolyzed with the highest SprP activity of ≥200 AU/min were used to create a preferred sequence motif (Schneider and Stephens 1990; Crooks and Stephens 2004).

### Protein identification

Proteins were identified by matrix-assisted laser desorption/ionization time-of-flight mass spectrometry (MALDI-TOF-MS) according to the protocol described by Schaffer et al. (2001). Briefly, protein bands were cut out of an SDS-PAGE gel and washed several times with 350 *μ*L 0.1 mol/L NH_4_HCO_3_ (in 30% (v/v) acetonitrile) until the gel slice was colorless and subsequently dried in a vacuum centrifuge. For tryptic digestion, the gel slice was incubated for 16 h with 6 *μ*L of a trypsin solution (10 ng/*μ*L, in 3 mmol/L Tris-HCl, pH 8.8), trypsin was obtained from Promega (USA). Digested peptides were dissolved by adding 2 *μ*L A. dest and 5 *μ*L 30% (v/v) acetonitrile with 0.1% (w/v) trifluoroacetic acid and incubated for 15 min in an ultra-sonification water bath. Subsequently, 1 *μ*L sample was spotted on Prespotted AnchorChip 96 (Bruker, Bremen, Germany) and washed after 3 min incubation with 7 *μ*L 10 mmol/L (NH_4_)_3_PO_4_. Peptide masses were determined with an UtraflexIII system (Bruker, Bremen, Germany) and database search was performed with MASCOT (Perkins et al. 1999).

## Results

### Production and purification of functional SprP

**Figure 1:**
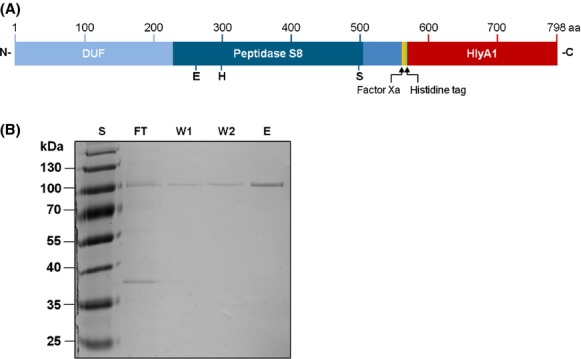
Expression in *Escherichia coli* and purification of SprP. (A) Schematic representation of the SprP-HlyA1 fusion protein used for expression and secretion in *E. coli via* the type I secretion system; SprP (blue) is shown with the DUF, Peptidase S8 domain, and the short C-terminal extension. Putative active site residues located within the Peptidase S8 domain are indicated. HlyA1 secretion signal (red) was fused to the C-terminus of SprP. Additionally, a six residue histidine tag (green) and a factor Xa recognition site for cleavage of the secretion signal (yellow) were inserted. (B) Analysis of SprP-HlyA1 fusion protein isolated from *E. coli* culture supernatants. Ten microliter each of different fractions obtained after chromatography on a Ni-NTA column were analyzed by SDS-PAGE and subsequent staining with Coomassie Brilliant Blue. The theoretical M_r_ of the SprP fusion protein is 88 kDa. Lanes show fractions of FT, first (W1) and second (W2) washing step with Tris-HCl buffer containing 20 and 30 mmol/L imidazole, respectively, and final elution with Tris-HCl buffer containing 250 mmol/L imidazole (E). S = M_r_ standard proteins (PageRuler Plus Prestained Protein Ladder, Fermentas, Sankt Leon-Rot Germany), aa = numbering of amino acids. DUF, domain of unknown function; SDS-PAGE, sodiumdodecyl sulfate polyacrylamide gel electrophoresis; FT, flow through.

Numerous attempts to express functional SprP in *E. coli*, *Pseudomonas putida* and *P. aeruginosa* resulted in accumulation of the recombinant protein as insoluble and enzymatically inactive inclusion bodies (data not shown). Finally, we decided to try a novel expression system where the recombinant protein is fused to a hemolysin secretion signal and is subsequently secreted *via* the type I secretion system (T1SS) of *E. coli* (Schwarz et al. 2013). Here, the fusion protein consisted of SprP fused to a 23 kDa HlyA1 secretion signal obtained from hemolysin A, an internal histidine tag, and a recognition site for factor Xa protease (Fig.1A). The native signal sequence of SprP was deleted to ensure secretion exclusively *via* T1SS. For the production and secretion of the fusion protein, *E. coli* cells harbored plasmids pSU-SprPminLS encoding the fusion protein and pK184-HlyBD encoding the transport proteins hemolysin B and D which, in combination with the constitutively expressed TolC protein, build the functional T1SS. Cell-free culture supernatant of the expression culture was used for SprP-HlyA1 purification and analyzed by SDS-PAGE (Fig.1B). The eluted protein was identified as the SprP-HylA1 fusion protein by MS (data not shown). This purification method resulted in a yield of 0.6 mg pure SprP protein per 100 mL supernatant.

### Posttranslational processing is required for SprP activation

**Figure 2:**
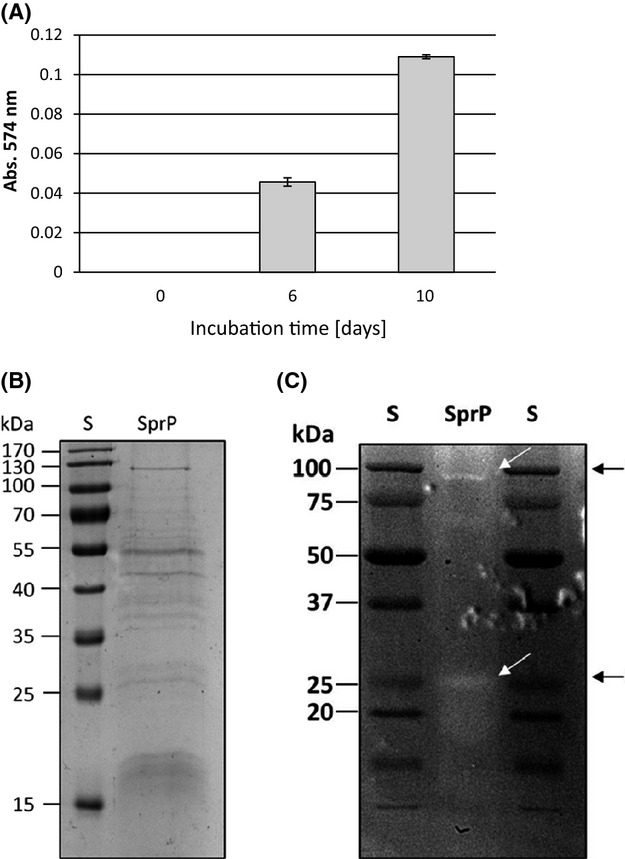
Kinetics of SprP activation. (A) Purified SprP-HylA1 fusion protein was incubated at 8°C and enzymatic activity tested with resorufin-labeled casein as the substrate. (B) SprP in 200 mmol/L Tris-HCl, 5 mmol/L CaCl_2_, pH 8 was 10-fold concentrated by TCA precipitation and a 10 *μ*L aliquot was analyzed by SDS-PAGE; S = M_r_ standard proteins (PageRuler Plus Prestained Protein Ladder, Fermentas, Germany). (C) One microgram of enzymatically active SprP was subjected to casein gel zymography. White arrows indicate proteins showing protease activity corresponding to M_r_ of 88 kDa (SprP-HlyA1 fusion protein) and 26 kDa (mature SprP). S = M_r_ standard proteins (Precision Plus Protein Dual Color Standard, Bio-Rad, Germany). TCA, trichloroacetic acid; SDS-PAGE, sodiumdodecyl sulfate polyacrylamide gel electrophoresis.

Purified SprP-HlyA1 fusion protein did not show protease activity (data not shown). Since it is known that proteases are often synthesized as inactive precursors which need to be activated by posttranslational processing (Khan and James 1998), the inactive SprP was incubated at 8°C and subsequently tested for activity. After 6 days of incubation, an increase in proteolytic activity was detected reaching a maximum after 10 days (Fig.2A). Raising of the incubation temperature to 37°C or incubation with factor Xa protease did not result in faster activation of the enzyme (data not shown). SDS-PAGE analysis of the active fraction revealed multiple bands (Fig.2B); subsequent zymography identified two protein bands with enzymatic activity. These bands which correspond to the predicted protein sizes represent the SprP-HlyA1 fusion protein of M_r_ 88 kDa and the mature and enzymatically active SprP of M_r_ 26 kDa (Fig.2C) as confirmed by subsequent MALDI-TOF-MS analysis. The additional protein bands with M_r_ ∼50 and ∼70 kDa also contained peptides originating from SprP suggesting that they represent intermediates resulting from autocatalytic processing of SprP-HlyA1.

By HPLC (High-performance liquid chromatography) size exclusion chromatography, proteolytic activity was exclusively detected in a fraction with a retention time of 8.89 min (Fig. S1). From a calibration curve obtained with the standard proteins bovine serum albumin, albumin, and *α*-chymotrysin, an apparent molecular weight of 26 kDa was calculated for SprP thus supporting the results obtained by zymography.

### Biochemical properties of SprP

**Figure 3:**
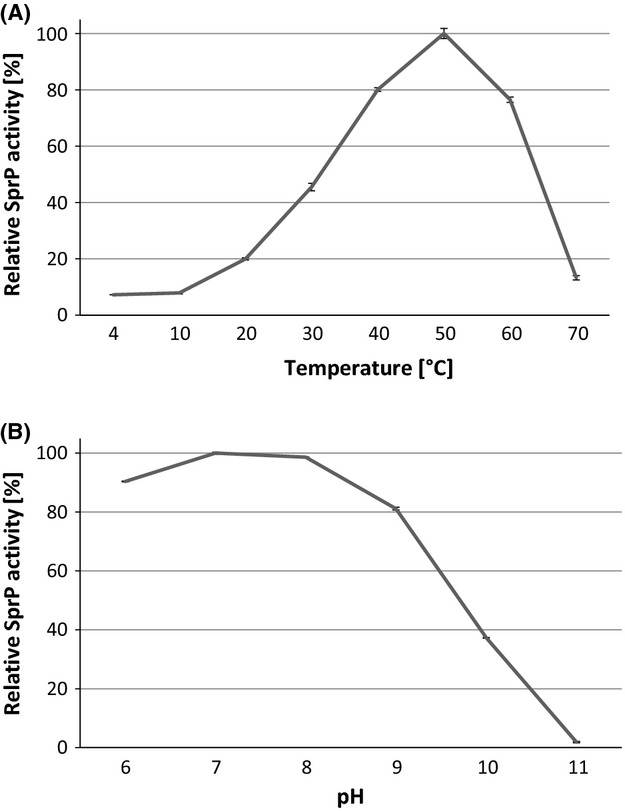
Temperature- and pH- profiles of SprP. Proteolytic activity was determined with resorufin-labeled casein as the substrate at (A) temperatures ranging from 4°C to 70°C and (B) pH ranging from 6 to 11. Highest activities were arbitrarily set as 100%. Graphs represent average values and standard deviations of triplicate determinations.

**Figure 4:**
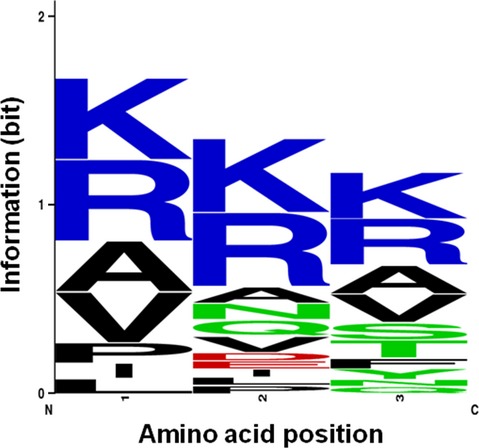
Preferred amino acid sequence motifs hydrolyzed by SprP. SprP preferentially hydrolyzed tripeptide substrates containing basic (blue) and hydrophobic (black) residues. At amino acid positions 2 and 3, acidic (red) and polar/uncharged residues (green) were also tolerated. Sequence logo was generated as described (Schneider and Stephens 1990; Crooks et al. 2004).

Biochemical properties were determined by using a self-processed and enzymatically active SprP (pre-incubated for 10 days, Fig.2A) which consists of a mix of SprP-HlyA1 fusion protein and native SprP. The enzymatic activity of SprP was tested within the temperature range of 4–70°C and pH range 6–11. The optimum temperature was determined as 50°C (Fig.3A) and a broad pH optimum between pH 7 and 8 was observed (Fig.3B). In addition, a library consisting of 3375 synthetic peptides was screened to characterize SprP substrate specificity. The results showed a strong preference of SprP for the hydrolysis of basic and hydrophobic tripeptides (Fig.4). Furthermore, the inhibition of SprP activity by specific protease inhibitors was tested. Only the serine protease-specific inhibitors AEBSF and TPCK were capable to significantly reduce SprP activity with AEBSF resulting in about 76% and TPCK in about 26% inhibition. In contrast, the metallo-, cysteine- and aspartyl-specific protease inhibitors EDTA, E-64, and pepstatin A did not inhibit SprP activity (Table2).

**Table 2:** Inhibition of SprP activity by protease inhibitors

Inhibitor	Specificity	Concentration (mmol/L)	Inhibition (%)
Control	–	–	0.0 ± 3.7
AEBSF	Serine protease	2	75.7 ± 1.9
TPCK	Serine protease	2	25.8 ± 3.1
EDTA	Metallo protease	7	0.0 ± 3.4
E-64	Cysteine protease	0.1	6.2 ± 2.0
Pepstatin A	Aspartyl protease	0.1	6.0 ± 1.1

SprP (3 *μ*g) was incubated in 200 mmol/L Tris-HCl, 5 mmol/L CaCl_2_, pH 8 buffer for 1 h at 4°C in the presence of the respective inhibitor. Protease activity was determined with resorufin-labeled casein as the substrate. The enzymatic activity of a control reaction without protease inhibitor was set as 100%. AEBSF, 4-(2-aminoethyl)benzenesulfonyl fluoride hydrochloride; TPCK, *N*-*p*-tosyl-l-phenylalanine chloromethyl ketone; EDTA, ethylenediaminetetraacetic acid; E-64, *N*-(trans-epoxysuccinyl)-l-leucine 4-guanidinobutylamide; pepstatin A, Iva-Val-Val-Sta-Ala-Sta.

### The sprP promoter activity is induced by growing *P. aeruginosa* at higher temperature

**Figure 5:**
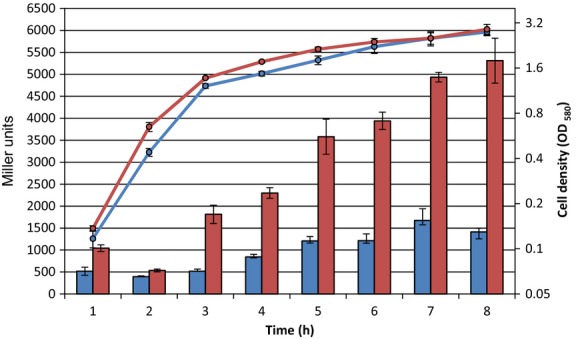
Promoter activity of *sprP* in *Pseudomonas aeruginosa* cultivated at 37°C and 43°C. *Pseudomonas aeruginosa* harboring plasmid pTZ110 as a control and pTZ*sprP* were cultivated at 37°C (blue) and 43°C (red) and *β*-galactosidase activity (bars) was determined according to the method of Miller (1972). Bacterial growth curves were determined by measuring the optical density as OD_580 nm_. Graphs represent average values and error bars indicate standard deviations from triplicate determinations.

*Pseudomonas aeruginosa* can grow at temperatures ranging from 25°C to 42°C (Tsuji et al. 1982) and is usually cultured under laboratory conditions at 37°C. The observation that the SprP temperature optimum of 50°C significantly exceeded the optimum growth temperature of *P. aeruginosa* prompted us to investigate the influence of the growth temperature on *sprP* promoter activity. To this end, a *lacZ* reporter gene fusion with the native *sprP* promoter DNA region was tested in *P. aeruginosa* PAO1. *Pseudomonas aeruginosa* cultures were cultivated for 8 h at 37°C and 43°C, respectively, and cell growth and *β*-galactosidase activity were determined. Whereas no significant differences in growth were observed for both *P. aeruginosa* cultures, *β*-galactosidase activities started to increase after 3 h of growth and reached a 3.8-fold higher activity after 8 h for the culture grown at 43°C as compared to the culture grown at 37°C (Fig.5).

## Discussion

The protease SprP is the second subtilase reported to be produced by *P. aeruginosa* PAO1. Recently, we have shown that a *P. aeruginosa* Δ*sprP* strain exerts a pleiotropic phenotype suggesting that SprP is a regulatory protease (Pelzer et al. 2014). In this study, we describe the purification and biochemical characterization of SprP which, as a first step, required its functional expression. Numerous attempts in *P. aeruginosa* and the heterologous hosts *E. coli* and *P. putida* including variation in growth media and temperature always resulted in the formation of catalytically inactive inclusion bodies (data not shown). Finally, we decided to try expression of *sprP* in *E. coli* with subsequent secretion using a newly constructed system (Schwarz et al. 2013). A SprP-HlyA1 fusion protein was constructed and expressed simultaneously with proteins forming the T1SS (Fig.1A). Here, secreted proteins fold only after secretion in the extracellular space thus avoiding the formation of intracellular proteolytic activity which may destroy important cellular functions. Furthermore, the formation of inclusion bodies is less favored in the culture supernatant because more space is available which reduces the probability of interactions between unfolded protease molecules. Apparently, these conditions promoted the formation of stable and soluble SprP that could subsequently be purified from the culture supernatant as shown in Figure1B. We observed that a significant fraction of the SprP-HlyA1 fusion protein did not bind to a Ni-NTA column, probably because of a reduced accessibility of the internal histidine tag (data not shown).

Most subtilases are produced as inactive zymogens that need autocatalytic processing of a prodomain for activation (Ikemura et al. 1987; Ikemura and Inouye 1988; Siezen and Leunissen 1997). Initially, the purified SprP fusion protein did not show proteolytic activity. However, activity appeared after prolonged incubation at 8°C suggesting that autocatalytic activation is also needed for SprP. SDS-PAGE analysis of the enzymatically active SprP fraction revealed multiple protein bands of M_r_ 17–130 kDa (Fig.2B) and the prominent band representing the SprP-HlyA1 fusion protein (Fig.1B) disappeared. Thus, we conclude that limited proteolysis occurred and resulted in SprP activation.

Casein gel zymography revealed a molecular weight of about 26 kDa for mature enzymatically active SprP (Fig.2C). The zymogram also showed low proteolytic activity of an 88 kDa protein presumably representing the SprP-HlyA1 fusion protein thus indicating incomplete processing. Both bands were identified as SprP by MALDI-TOF-MS. A molecular weight of about 26 kDa for mature SprP was also determined by HPLC SEC thereby confirming the result obtained by zymography (Fig. S1). The typical subtilase M_r_ ranges from 18 to 90 kDa with many members having a molecular weight of 27 kDa (Maurer 2004). Presently, we assume that both the DUF and the C-terminal domain are cleaved during processing, that is, mature SprP would consist only of the Peptidase S8 domain lacking the His-tag and thus would not bind to a Ni-NTA column as we observed during purification. The substrate preference of SprP for basic and hydrophobic tripeptides (Fig.4) suggests several putative sites for autoproteolytic processing. However, if amino acids forming the catalytic triad in the Peptidase S8 domain are excluded, two sites for hydrolysis remain; one located at the C-terminus of the DUF (YYQ RRV RAR QAP) and the other 29 amino acids downstream of the catalytic active serine in the Peptidase S8 domain (MLL RRS AMT). Cleavage at these sites would result in peptides of Mr ∼25 kDa representing the DUF and of Mr ∼30 kDa representing the Peptidase S8 domain which roughly corresponds to the observed enzymatically active enzyme of Mr ∼26 kDa.

Until now, the classification of SprP is based on its homology to subtilases, the capability to hydrolyze a serine protease substrate, and by the loss of activity upon deleting the predicted active serine residue (Pelzer et al. 2014). Here, we have used specific protease inhibitors to further characterize SprP as a serine protease. The activity of SprP was strongly reduced by AEBSF and TPCK. AEBSF leads to the sulfonation of the hydroxyl group of the active site serine and thus inhibits the activity of serine proteases. TPCK is a chymotrypsin substrate analog and inhibits serine proteases by irreversible binding of histidine in the active site (Schoellmann and Shaw 1963; Powers et al. 2002). The cysteine protease inhibitor E-64 and the aspartyl protease inhibitor pepstatin A were unable to reduce the SprP activity significantly as was EDTA which complexes metal ions like Ca^2+^ (Table2). Many subtilases need Ca^2+^ ions as cofactors to increase their stability (Alexander et al. 2001). Our results confirm the classification of SprP as a serine protease and, furthermore, indicate that Ca^2+^ ions are not needed for SprP activity.

Additionally, we have determined temperature and pH profiles of mature SprP. The enzyme showed highest activity in a temperature range of 40–60°C with a maximum at 50°C and at a pH range of 6–9 with a maximum at 7–8 (Fig.3A and B). These characteristics fit with the majority of subtilases which show highest activity at neutral pH and are often thermostable (Rawlings et al. 2012). Further studies are needed to determine the complete amino acid sequence of mature SprP as well as putative factors involved in initiating autocatalytic cleavage.

The comparative analysis of the *sprP* promoter activity at 37°C and 43°C revealed a strong increase in expression at the higher temperature (Fig.5). Proteases as well as chaperones are part of the bacterial cellular protein quality control system and their expression often coincides with the heat shock response (Arsene et al. 2000). In bacteria, ATP-dependent proteases often catalyze the degradation of denatured proteins (Gottesman 1996; Sauer et al. 2004). In addition, ATP-independent intracellular proteases like periplasmatic DegP from *E. coli* are part of the protein quality control system (Clausen et al. 2002). Similar to *sprP*, *degP* expression is also induced under heat shock and DegP shows high-proteolytic activity above 40°C (Lipinska et al. 1988; Connolly et al. 1997; Spiess et al. 1999).

Furthermore, we observed a preference of SprP for hydrolysis of basic and hydrophobic amino acid sequences. Such residues are known to destabilize proteins when present on the protein surface where they can function as a signal for regulated proteolysis (Mogk et al. 2007). These observations together with our recent data on pleiotropic effects exerted by SprP (Pelzer et al. 2014) suggest an important role for this protease in a regulatory circuit involved in stress response of *P. aeruginosa*.

## Supporting Information

**Figure S1.** HPLC SEC analysis of enzymatic active SprP. (A) Purified and active SprP was subjected to HPLC SEC, and the absorbance of eluted fractions was monitored at 220 nm and 280 nm. Standard proteins were thyroglobulin (670 kDa), bovine serum albumin (67 kDa), albumin (45 kDa), chymotrypsinogen (25 kDa), and uridine (0.2 kDa) dissolved in buffer. (B) Fractions 1, 3, and 7 (see A) were analyzed for protease activity with resorufin labeled casein as the substrate by determination of the absorbance at 574 nm (Abs_574 nm_). AU, arbitrary unit.
