# Supplementary material for: Functional expression, purification, and biochemical properties of subtilase SprP from Pseudomonas aeruginosa
Source: Microbiologyopen. 2015 Jul 14;4(5):743–52. doi: 10.1002/mbo3.275 (PMC4618607; doi:10.1002/mbo3.275)
Supplement: Supplementary file 1 — Figure S1. HPLC SEC analysis of enzymatic active SprP. (A) Purified and active SprP was subjected to HPLC SEC, and the absorbance of eluted fractions was monitored at 220 nm and 280 nm. Standard proteins were thyroglobulin (670 kDa), bovine serum albumin (67 kDa), albumin (45 kDa), chymotrypsinogen (25 kDa), and uridine (0.2 kDa) dissolved in buffer. (B) Fractions 1, 3, and 7 (see A) were analyzed for protease activity with resorufin labeled casein as the substrate by determination of the absorbance at 574 nm (Abs574 nm). AU, arbitrary unit. [file mbo30004-0743-sd1.docx]

**Supplementary Material**

*Material and Methods*

*HPLC size exclusion chromatography*

The apparent molecular weight of SprP was determined by HPLC (LC-10Ai, Shimadzu, Duisburg, Germany) size exclusion chromatography on a Biosep-SEC S2000 column with 300 x 7.8 mm dimension (Phenomenex Ltd., Aschaffenburg, Germany) and a photodiode array detector. The system was equilibrated with 200 mM Tris buffer, pH 7.5 containing 5 mM CaCl_2_ .Standard proteins thyroglobulin (670 kDa), bovine serum albumine (67 kDa), albumine (45 kDa), chymotrypsinogen (25 kDa) and uridine (0.2 kDa) were dissolved in buffer and used for calibration. The column was loaded with purified SprP (0.1 mg dissolved in buffer), spectra at 220 nm and 280 nm were determined, and fractions of 500 µl were collected and tested for protease activity.

*Results*

**Figure S1**:

(**A**)


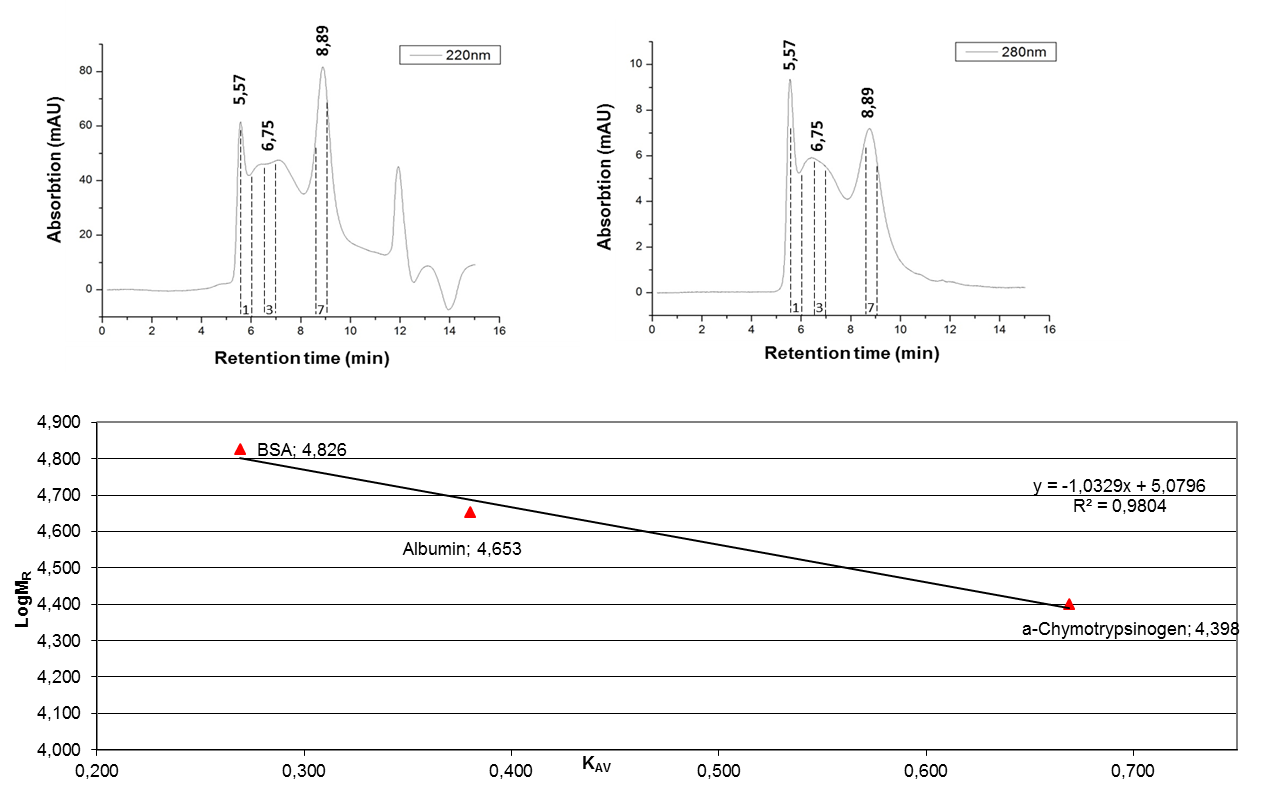


(**B**)

**7**

**1**

**3**

**Fraction no.**

**Fig. S1: HPLC SEC analysis of enzymatic active SprP. (A):** Purified and active SprP was subjected to HPLC SEC and the absorbance of eluted fractions was monitored at 220 nm and 280 nm. Standard proteins were thyroglobulin (670 kDa), bovine serum albumin (67 kDa), albumin (45 kDa), chymotrypsinogen (25 kDa) and uridine (0.2 kDa) dissolved in buffer. **(B):** Fractions 1, 3, and 7 (see panel A) were analyzed for protease activity with resorufin labeled casein as the substrate by determination of the absorbance at 574 nm (Abs_574 nm_). AU = arbitrary unit.
